# Uni-Directionally Oriented Fibro-Porous PLLA/Fibrin Bio-Hybrid Scaffold: Mechano-Morphological and Cell Studies

**DOI:** 10.3390/pharmaceutics14020277

**Published:** 2022-01-25

**Authors:** Andrew F. Uehlin, Jeremy B. Vines, Dale S. Feldman, Elijah Nyairo, Derrick R. Dean, Vinoy Thomas

**Affiliations:** 1 Department of Materials Science and Engineering, University of Alabama at Birmingham, Birmingham, AL 35205, USA; auehlin@gmail.com; 2 Department of Biomedical Engineering, University of Alabama at Birmingham, Birmingham, AL 35205, USA; jbvines@gmail.com (J.B.V.); dfeldman@uab.edu (D.S.F.); 3 Biomedical Engineering, Alabama State University, Montgomery, AL 36101, USA; enyairo@alasu.edu

**Keywords:** electrospinning, ligament, fibrin scaffold, biohybrid scaffold, ACL, polymer nanofiber

## Abstract

In this study, we report a biohybrid oriented fibrous scaffold based on nanofibers of poly(l-lactic acid) (PLLA)/fibrin produced by electrospinning and subsequent post-treatment. Induced hydrolytic degradation of the fibers in 0.25 M NaOH solution for various time periods followed by the immobilization of fibrin on the hydrolyzed fiber surfaces was shown to significantly affect the mechanical properties, with the tensile strength (40.6 MPa ± 1.3) and strain at failure (38% ± 4.5) attaining a value within the range of human ligaments and ligament-replacement grafts. Unidirectional electrospinning with a mandrel rotational velocity of 26.4 m/s produced highly aligned fibers with an average diameter of 760 ± 96 nm. After a 20-min hydrolysis treatment in NaOH solution, this was further reduced to an average of 457 ± 89 nm, which is within the range of collagen bundles found in ligament tissue. Based on the results presented herein, the authors hypothesize that a combination of fiber orientation/alignment and immobilization of fibrin can result in the mechanical and morphological modification of PLLA tissue scaffolds for ligament-replacement grafts. Further, it was found that treatment with NaOH enhanced the osteogenic differentiation of hMSCs and the additional inclusion of fibrin further enhanced osteogenic differentiation, as demonstrated by decreased proliferative rates and increased ALP activity.

## 1. Introduction

Mimicking the morphological and mechanical properties of native tissue is a key element of tissue engineering scaffolds. Producing aligned fibers mimetic of collagen bundles in size, orientation, and mechanical function is desired for ligament engineering applications. Electrospinning is a technique used to create fibrous polymer scaffolds with fiber diameters ranging from the micro- to nanoscale, depending on experimental parameters [1,2,3,4]. Accordingly, for the purpose of mimicking oriented fiber morphology and for increased mechanical properties, the fibers can be collected on a rotating mandrel, as opposed to a stationary frame [3]. The fiber morphology and orientation in electrospun polymers are relevant for the mechanical strength of various tissue scaffolds. Similarly, the aligned orientation of the fibers can have an influence on the growth and orientation of cells cultured in vitro [3]. Past studies indicate that fibroblasts cultured on aligned polyurethane nanofibers secreted more extracellular matrix (ECM) collagen than fibroblasts on randomly oriented fibers [5]. Furthermore, studies have shown that when mechanical stimuli were applied along the alignment axis, increased ECM production by the fibroblasts was also observed [5].

Studies have shown that PLA can potentially have suitable characteristics for ligament engineering, with material properties that can be tailored to obtain function and design flexibility desirous in a scaffold [6,7,8]. Due to its hydrophobic nature and smooth morphology, the surface of PLA alone is not an ideal substrate for cellular integration and the subsequent synthesis of new tissue. Surface functionalization can be induced by NaOH to modify the morphology and surface chemistry of the PLA [9,10,11,12,13,14,15,16,17,18,19,20,21,22,23,24,25,26,27,28,29,30,31]. This type of degradation can also lower mechanical properties through hydrolytic chain scission, leading to a reduction in molecular weight and a subsequent linear reduction in bulk area. Additionally, it aids bioactivity through the production of carboxylic acid groups on the PLA surface, allowing for more effective protein immobilization and improved cell activities, such as adhesion and proliferation [12,13]. The use of NaOH to introduce carboxyl groups and increase surface roughness can potentially assist in the effective binding of cells [9], indicating that the combination of the surface carboxyl groups with an intermediary protein (fibrin) has a potent effect on cellular attachment and proliferation. Biological materials such as hyaluronic acid [14], silk [15], collagens and fibrin [16] have been explored as alternatives to collagen bundles of ligament tissue.

Fibrin is a natural polymer in the human body that is critical for hemostasis and wound healing. Fibrin can be created ex vivo through the rapid enzymatic polymerization of fibrinogen with thrombin from either allogeneic or autologous sources [17]. Due to a natural binding affinity, the immobilization of many growth factors as well as improved cell seeding efficiency and uniformity of cell distribution is possible [18,19,20]. Furthermore, the biocompatibility and ease of processing from autologous sources eliminate immunological concerns [21].

The mechanical properties of fibrin have been evaluated in previous studies [22,23,24]. Elastic moduli were determined on fibrin fibers using laser tweezers in a phosphate-buffered saline (PBS) environment [24]. The moduli were calculated at 14.5 and 1.7 MPa in the crosslinked and uncrosslinked states, respectively. A combination of atomic force microscopy (AFM) and fluorescence microscopy was used to measure the strain at failure (extensibility) [23]. The strain at failure was surprisingly high, with 332% and 226% elongation in the crosslinked and uncrosslinked states, respectively. These properties of fibrin may affect the overall mechanical resilience of poly(l-lactic acid) (PLLA) when immobilized on the surface.

In this study, unidirectional electrospinning was used to create highly aligned nanofibers of PLLA, and then NaOH hydrolysis of the PLLA nanofibers was induced over various time periods. The degree of fiber alignment from the unidirectional electrospinning, as well as the effects of NaOH hydrolysis on the fiber alignment, fiber diameter, surface morphology and immobilization of fibrin, was examined using SEM. The effects of unidirectional electrospinning and induced hydrolytic degradation on crystallinity were assessed via differential scanning calorimetry (DSC). Additionally, the physical effects of NaOH hydrolysis on in vitro swelling of the PLLA nanofibers were investigated. Furthermore, studies to evaluate human mesenchymal stem cells’ (hMSCs) behavior on the various conditions of the PLLA nanofibers were performed, particularly regarding proliferation and differentiation. The goal was to determine the relationship of processing parameters to morphology as well as the preliminary cellular activity of hMSCs for the ligament-bone interface.

## 2. Materials and Methods

### 2.1. Production of an Aligned Electrospun PLLA Nanofiber Mats 

**Figure 1:**
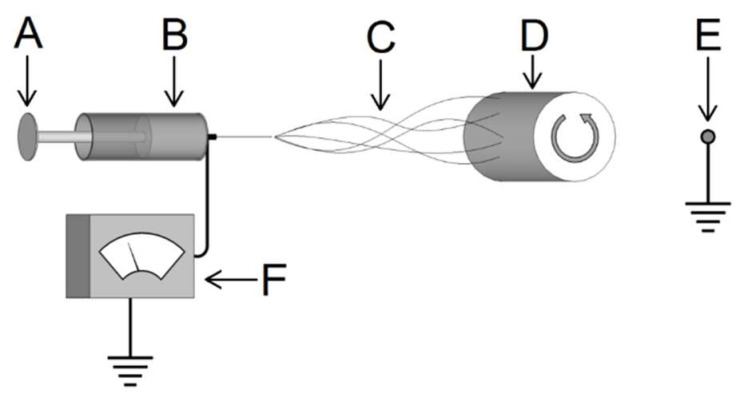
Schematic of electrospinning system consisting of: A—syringe fed by a pump with a charged lead from the voltage source to the needle, B—polymer solution within the syringe, C—ejected polymer forming micro- or nanofibers, D—rotating mandrel collecting the fibers, E—grounding point behind the rotating mandrel, and F—a variable DC voltage supply.

A 20% *w*/*v* solution of PLLA (Medisorb 100 L Poly(l-Lactide) Lakeshore Biomaterials, Birmingham, AL, USA) using 1,1,1,3,3,3-hexafluoro-2-propanol (HFIP) as a solvent was prepared. The PLLA/HFIP solution was transferred to a 10 mL syringe. Unidirectional electrospinning equipment was used (Figure 1); the syringe was affixed to a 20 G × 20 cm septum penetrating needle (M-4855, Popper & Sons, Inc., New Hyde Park, NY, USA) connected to a DC high voltage source (M826, Gamma High Voltage Research, Ormond Beach, FL, USA) which was held constant at 15 kV. The flow rate was maintained by a syringe pump (Fusion 100, Chemyx, Inc., Stafford, TX, USA) and set at 1 mL/h for 2 h. Fibers were collected at a distance of 15 cm on a 15 cm × 25 cm sheet of aluminum foil attached via cellophane tape to a cylindrical aluminum mandrel 67 cm in diameter and 15.5 cm in length, rotating at 7000 rpm (24.6 m/s), with a stationary grounding point 3.5 cm behind the mandrel. Placing the grounding point behind the mandrel (as opposed to grounding the mandrel itself) was found to yield fiber uptake over a narrower and more consistent area (approximately 9 cm width perpendicular to the rotation axis compared to 15 cm using a grounded mandrel) (Figure 1).

**Figure 2:**
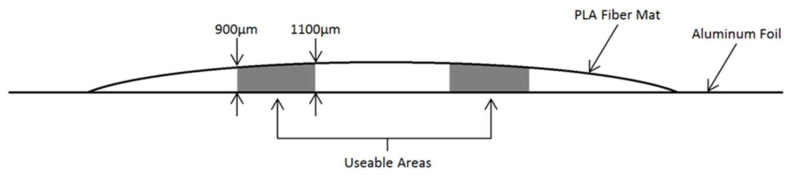
Cross-sectional representation of electrospun PLA mat depicting thickness 900–1100 µm areas selected for use throughout the study.

Due to the random deposition of fibers during the electrospinning process, the thickness of the fiber mat was inconsistent over the approximate 9 cm wide fiber uptake area, with a greater thickness towards the center of the samples. Multiple thickness measurements were taken across the transverse direction of the aligned fibers from each sample (without the foil) using a thermomechanical analyzer (TMA) (Model Q-400, TA Instruments, New Castle, DE, USA) [3]; only the areas with a thickness range of 900–1100 µm were used throughout experimentation (Figure 2). This resulted in two useable sections per PLLA mat, approximately 20 cm × 2 cm. Multiple PLLA mats were produced in this manner to accommodate the sample quantity needed throughout this investigation.

The PLLA fiber mats were cut into 5 mm × 45 mm sections, with the 45 mm length parallel to the alignment of the fibers. Following any surface treatment performed, the thickness of each sample was measured in three different locations along the sample width by the TMA and averaged to calculate cross-sectional area for tensile testing [3,9].

### 2.2. Production of Electrospun PLLA Mats with Altered Mandrel Velocity

For the purpose of evaluating mandrel rotation velocity on fiber diameter, alignment, and crystallinity, two additional PLLA fiber mats were produced using the same parameters with the exception of an altered mandrel rotation speed of 0 rpm (stationary) and 3500 rpm (12.3 m/s), respectively. These two PLLA mats were fabricated as part of optimizing the fiber alignment and did not undergo any of the subsequent NaOH treatments described below.

### 2.3. Surface Treatment by Controlled NaOH Hydrolysis

**Scheme 1:**
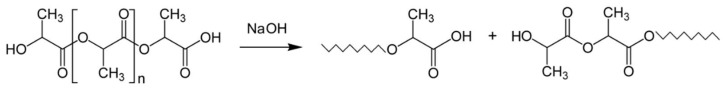
Surface hydrolysis of PLLA treated with NaOH showing ester group lysis.

Samples were placed into a glass beaker containing an aqueous solution of 0.25 M NaOH at room temperature (21 °C). The samples were immersed for 1, 5, 10, 20, 30 and 40 min. A possible surface reaction is given in Scheme 1. Five samples were processed at a time and monitored to ensure complete immersion at all times and prevent clinging to each other and the beaker wall. After their respective treatment times, the samples were removed from the NaOH solution and immediately immersed in a 500 mL beaker of deionized water to dilute any remaining NaOH solution. The samples were then rinsed 3 times with deionized water, placed on a low-lint paper cloth and dried in a desiccator for 72 h. 

### 2.4. Biohybrid Scaffolds by Immobilization of Fibrin onto Electrospun PLLA

Utilizing previously employed methods [9], the combination of bovine thrombin (T9549, Sigma Aldrich Co., St. Louis, MO, USA) and bovine fibrinogen (F8630, Sigma Aldrich Co., St. Louis, MO, USA) resulted in the self-assembly of a homogenous surface layer of fibrin on the PLLA fiber samples. This method was used on a portion of the 20 min NaOH-treated samples, selected as a result of the modified fiber diameter, surface morphology, and cellular response, determined by the authors [9].

Modifying methods from previous studies to produce a homogenous surface layer of fibrin on a variety substrate [25,26], 50 mL of an aqueous solution containing 1.1 U/mL of bovine thrombin (T9549, Sigma Aldrich Co., St. Louis, MO, USA) and 5 mM of calcium chloride was prepared and immersed in a water bath at 37 °C. Similarly, a 50 mL aqueous solution containing 4 mg/mL of bovine fibrinogen (F8630, Sigma Aldrich Co., St. Louis, MO, USA) and 50 mM of HEPES-buffered saline was prepared and immersed in a water bath at 37 °C. When combined, the thrombin/fibrinogen solution was observed to fully polymerize into fibrin within 15 min. To effectively immobilize fibrin on the surface of the PLLA fiber mats, the thrombin and fibrinogen solutions were combined; this was immediately followed by immersion of the glass slides with the PLLA. After a few seconds, the slides were removed and placed in an incubator at 37 °C for 15 min, allowing the thrombin and fibrinogen to self-assemble into fibrin on the exposed surface of the PLLA mat. The slides were then triple rinsed with PBS to remove unbound/excess fibrin, then placed in a desiccator overnight to dry.

### 2.5. Mechanical Testing of the PLLA Nanofiber Mats

A micromechanical testing system (Minimat Model 2000, Rheometrics, Piscataway, NJ, USA) was used on the various samples to establish mechanical behavior under uniaxial tension. The distance between the clamps was set at 25 mm. Sections of adhesive-backed 320 grit sandpaper were affixed to the contact surfaces of the test clamps to provide better contact and minimize slipping of the bulk fibers under load. The samples were placed in the clamps and centered with calipers (±0.25 mm) to ensure uniaxial loading along the fiber alignment direction prior to tightening the clamps. A heated chamber was used for some of the samples to establish mechanical properties at temperatures up to 37 °C (normal human body temperature). Testing was performed in tension mode with a load cell of 200 N and at a strain rate of 5 mm/min until failure. The mechanical properties of the samples were then defined based on these uniaxial data obtained.

### 2.6. Mechanical Properties of Wet/NaOH Treated Samples

Previous studies indicate up to 10% swelling of the NaOH-treated PLLA fiber mats when incubated at 37 °C in PBS solution (pH 7.4) for various times [9]. The study indicated that swelling of the PLLA mats was relatively consistent following one week of incubation, with minimal degradation. The effects of the swelling on the mechanical properties of the PLLA fibers were therefore evaluated by incubating half of the sample varieties in PBS at 37 °C for one week, followed immediately by mechanical testing.

### 2.7. Characterization of Fiber Alignment, Diameter, and Surface Morphology 

SEM was used to analyze fiber alignment, diameter, and morphology of the PLLA samples. Sections of the 7000 rpm, 3500 rpm, stationary (0 rpm), and various NaOH-treated samples were affixed to an SEM stub, sputter-coated with gold-palladium and observed under SEM (Quanta 650FEG, FEI Co., Hillsboro, OR, USA) at an accelerating voltage of 5 kV. The SEM micrographs were analyzed to measure fiber diameter utilizing image-analyzing software (Image-Pro Insight, Media Cybernetics, Silver Spring, MD, USA).

### 2.8. Assessment of Crystallinity by Differential Scanning Calorimetry (DSC)

DSC (Model-Q100, TA Instruments, New Castle, DE, USA) analyses were performed on PLLA samples of the following morphology: as received (pellet), thin film (cast from the electrospinning solution), unaligned electrospun fibers (0 rpm), aligned electrospun fibers (7000 rpm), and 20 min NaOH-treated aligned electrospun fibers. The degree of crystallinity for each sample was calculated using the following formula:

% Crystallinity = ((∆H_exp_)/(∆H_100_)) × 100

where ΔH_exp_ and ΔH_100_ are the melting enthalpy values for the experimental sample and a fully crystalline sample, respectively.

### 2.9. Swelling Studies

Owing to the decreased hydrophobicity of PLLA in the post-hydrolyzed condition, swelling of the hydrolyzed PLLA fibers is a consideration that requires investigation. To evaluate any physical changes that may occur under physiological conditions over time, the PLLA in the untreated and NaOH-treated conditions were placed in a phosphate-buffered saline (PBS) solution to evaluate swelling (PBS uptake) over various time periods. The PLLA fiber mat was cut into 1 cm^2^ samples, and the foil was removed. Half of the samples were subjected to the 20 min NaOH treatment protocol previously outlined. Samples of both conditions (untreated/NaOH treated) were placed into separate 25 mL glass vials containing PBS solution (pH 7.4), sealed, and incubated at 37 °C for 1, 7, 14, 30, and 60 days. At the end of each interval, the samples were removed from the PBS, dabbed on a lint-free paper towel to remove surface PBS, and immediately weighed to obtain wet mass. The samples were then placed in a desiccator for 72 h to dry thoroughly. The samples were removed from the desiccator and immediately weighed to identify dry mass. Percent swelling was calculated using the following equation:

Swelling (%) = [(M_w_ − M_d_)/M_d_] × 100

where M_w_ is the wet mass after the various incubation periods, and M_d_ is the mass after the sample has been dried. All samples were run in triplicate (*n* = 3) for statistical validity. 

### 2.10. Preparation of Fibrin Immobilized Samples for Cell Studies

The PLLA fiber mat was cut into 15 mm × 75 mm samples with the longer side parallel to the alignment of the fibers, and the foil was removed. Half of the samples were subjected to the 20 min NaOH treatment previously described. All samples (treated/untreated) were then placed on individual 25 mm × 75 mm glass slides, held in place by their static charge. Fibrin immobilization methods from previous studies to produce a homogenous surface layer of fibrin on a variety substrates [25,26] were carried out as described previously. This method was utilized with half of the aforementioned 20 min NaOH-treated samples for subsequent cell studies. Additional sections from the untreated and 20 min NaOH samples also underwent this technique and were then prepared for SEM as previously described. SEM micrographs were obtained prior to the cell studies to qualitatively assess surface morphology and binding efficacy of fibrin on PLLA in the untreated vs. NaOH-treated condition.

### 2.11. Sample Fixation to the Tissue Culture Plates

Due to their thin size (approximately 1000 µm), flexibility, and high static attraction to almost every surface in our laboratory, handling the PLLA samples without damaging them was very difficult and tedious. Additionally, once immersed in an aqueous cell culture environment, the samples would float to the top of the surface, fold onto themselves along the fiber alignment direction, or cling to the sides of the cell culture well, negating successful results of any cell study. Attempts at attaching the samples directly to the bottom of the well using tape or biocompatible adhesives also proved to be tedious and highly destructive of the samples. Previously, Focarete et al. [27] used Tecaflon rings fixed to the scaffolds using silicone rubber and Gualandi et al. [28] used Cell-Crown rings to hold PLLA scaffolds. To facilitate a consistent means of obtaining a flat, continuous, and undamaged sample surface at the bottom of the culture well, a unique sample fixation technique was implemented as described in Appendix A. The modification resulted in flat PLA mats affixed to the bottom of each of the wells with 1.26 cm^2^ of useable surface area. All of the prepared 24-well plates were placed under a UV hood for 2 h, followed by rinsing the wells with sterilized deionized water 12 h prior to seeding.

### 2.12. Human Mesenchymal Stem Cell (hMSC) Culture

The hMSCs were purchased (Lonza, Inc., Walkersville, MD, USA) and passaged, retaining only cells from passage numbers 4–6. The cells were cultured in mesenchymal stem cell basal medium (MSCBM) (Lonza, Inc., Walkersville, MD, USA) and supplemented with MSCBM SingleQuots (Lonza, Inc., Walkersville, MD, USA). Upon reaching confluence, cells were removed from the culture surface and deactivated by adding an equal volume of Dulbecco’s modified Eagle medium (DMEM) (Mediatech, Manassas, VA, USA) supplemented with 10% fetal bovine serum (FBS) (Thermo-Fisher Scientific, Watham, MA, USA), 1% amphotericin B, 1% penicillin,1% streptomycin (Mediatech, Manassas, VA, USA), and 1% l-glutamine. The hMSCs were then centrifuged at 1000 rpm for 5 min and re-suspended at a concentration of 15,000 cells per 677 μL of DMEM.

The solution was measured into 677 μL aliquots and transferred to the 24-well cell culture plates with the PE tubes prepared for the study. The 12-well cell culture plates were also prepared for sample imaging. Cell cultures were held constant under standard culture conditions (37 °C, 95% relative humidity, 5% CO_2_). Every 3–4 days, old media was replaced with 677 μL of fresh media. For the 24-well plate samples, the hMSCs were cultured, trypsinized and harvested at days 1, 7, and 14, then cryogenically stored in Eppendorf tubes at −80 °C until analysis of cellularity was performed and ALP activity was assessed.

### 2.13. Preparation of Samples for SEM Imaging

To characterize the morphology of the hMSCs in response to the various PLLA treatment conditions, separate samples were prepared to be observed by SEM imaging following cell culture. Unlike the samples prepared for analysis of cellularity, it was not essential that the PLLA cover the entirety of the well bottom since only the surface of the PLLA was observed for qualitative characterization of cell morphology. Samples of each of the PLLA treatment conditions were cut into 1 cm^2^ sections and affixed to a 15 mm diameter round glass coverslip (M-26021, Ted Pella Inc., Redding, CA, USA) using the same biocompatible adhesive used earlier (see Sample Fixation to the Tissue Culture Plates in Appendix A) and allowed to dry in a vacuum/desiccator overnight. The samples were then inserted face-up into sterile 12-well tissue culture plates and placed under a UV hood for 2 h for sterilization, followed by rinsing the wells with sterilized deionized water 12 h prior to seeding.

For the 12-well plate imaging samples, the samples were fixed using 2.5% glutaraldehyde and 2% paraformaldehyde in a sodium cacodylate buffer (0.2 M, pH 7.4) with deionized water. Following initial fixation, the samples were rinsed several times with PBS for a minimum of 15 min, followed by post-fixation with 1% sodium tetroxide in 0.1 M phosphate buffer for 1 h. After re-rinsing with PBS several times for 15 min, the samples were dehydrated using a series of graded ethanol: 70% for 15 min, 95% for 15 min, and 3 changes of 100% for 10 min each. The samples were then subjected to chemical drying using 2 parts 100% ethanol and 1 part hexamethyldisilazane (HDMS) (Electron Microscopy Sciences, Ft. Washington, PA, USA) for 15 min, 1 part 100% ethanol and 2 parts HDMS for 15 min, then 2 changes of 100% HDMS for 15 min each. All residual solution was aspirated from the samples, and then the samples were allowed to air-dry under a fume hood overnight. The samples were then affixed to a mount, sputter-coated, and placed in the SEM. Images were acquired at an accelerating voltage of 5 kV.

### 2.14. Analysis of Cellular Proliferation

Cellularity was analyzed using hMSCs harvested from the 24-well plates at days 1, 7, and 14. Picogreen assay (Molecular Probes, Eugene, OR, USA) was utilized to identify the double-stranded DNA content according to manufacturer specifications. 100 μL cell extracts were placed in 96-well plates for analysis. Picogreen dye was added into the sample preparations, then incubated in the dark for 15 min. Using a fluorescent microplate reader (Synergy HT, BIO-TEK Instruments, Winooski, VT, USA) filtered at 485/528 (EX/EM), the double-stranded DNA content was then measured and compared to a standard curve, correlating known DNA content.

### 2.15. Alkaline Phosphatase (ALP)Activity

Quantitative ALP activity was assessed on the hMSCS harvested from the 24-well plates at 14 days. Using a fluorimetric SensoLyte FDP Alkaline Phosphatase Assay Kit (Anaspec, San Jose, CA, USA), 50 μL aliquots of each sample were assayed for ALP content on a fluorescent microplate reader (using the same parameters as described in Analysis of Proliferation), then compared to a standard correlating known ALP content to fluorescence levels. A picogreen analysis was performed to normalize ALP expression via DNA content by determining the specific ALP activity present within each sample.

### 2.16. Statistical Analysis

The results presented herein are representative data sets with experiments performed using 6 samples (*n* = 6) for each condition at 1, 7, and 14 day time points. Values were expressed as ±standard error of the mean (S.E.M.). Using SPSS software (SPSS, Chicago, IL, USA), one-way analysis of variance (ANOVA) was performed to quantify any significant differences between conditions at each time point. Tukey multiple comparison tests were conducted to further determine significant differences between pairs. A value of *p* < 0.05 was considered significant for all tests.

## 3. Results and Discussion 

### 3.1. Mechanical Properties

PLLA nanofibers were produced utilizing electrospinning. Uniaxial tensile tests of the aligned electrospun PLLA fibers were performed using ten samples (5 dry, 5 wet) per surface treatment condition, including a control (unmodified aligned electrospun sample). The data revealed considerable variety in the mechanical properties based on the surface treatments employed on the samples. Surprisingly, the wet samples did not deviate beyond the range of the data obtained from the dry samples of each given condition. One-way ANOVA performed on the wet/dry samples for each treatment condition yielded *p*-values ranging from 0.12–0.28, indicating relatively equal sample means. Therefore, the data obtained for the wet and dry samples were combined. Numerical results are reported as mean ± standard error of the mean (S.E.M.) throughout this study.

**Figure 3:**
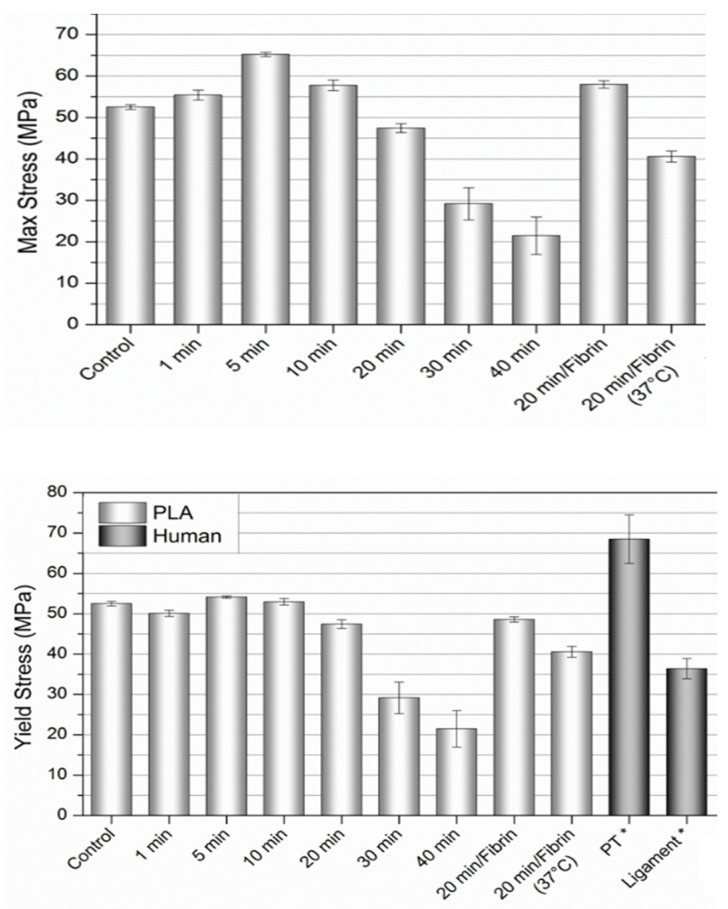
Tensile strength and yield strength of the aligned PLLA fibers with various surface treatments compared to natural ligament/graft tissue. Values expressed as mean ± S.E.M. (PT = patellar tendon, “ligament” includes ACL, PCL, and LCL). Data adapted with permission from Butler, et al., J. Biomech. 1986, 19, 425–432. [29]. Copyright Elsevier 2022.

The yield and maximum stresses for the samples are illustrated in Figure 3. The sample exposed to NaOH for 1 min indicated a slight loss of yield strength (50.1 MPa ± 0.78), while the 5 and 10 min exposure samples actually displayed slightly higher yield strength than the control sample, with values of 54.1 MPa ± 0.29, 53.0 MPa ± 0.79, and 52.5 MPa ± 0.59, respectively.

The variety of 20 min samples (20 min, 20 min + fibrin, and 20 min + fibrin at 37 °C) exhibited a modest decline in yield stress (47.4 MPa ± 1.1, 48.6 MPa ± 0.66, and 40.6 MPa ± 1.3, respectively), and displayed properties most similar to the ACL and ligament tissue data [29,30], particularly the 20 min + fibrin at 37 °C sample. The 30 and 40 min samples had a sharp decline in yield stress with a wider distribution of values (29.2 MPa ± 3.9 and 21.5 MPa ± 4.5, respectively). The maximum stress values were higher than the yield stress for the 1, 5, 10, and 20 min + fibrin samples (55.4 MPa ± 1.2, 65.2 MPa ± 0.48, 57.8 MPa ± 1.2, and 58.0 MPa ± 0.89, respectively).

The percent elongation at failure with the 1, 5 and 10 min NaOH-treated sample values were higher than the control sample (36.4% ± 1.6, 40.5% ± 1.8, 36.7% ± 1.7, and 29.2% ± 1.4, respectively) (see Supplementary Materials, Figures S1 and S2). The 20, 30 and 40 min samples (23.3% ± 3.3, 8.4% ± 0.60, and 6.5% ± 1.4, respectively) exhibited elongation at failure less than the control sample, with the 30 and 40 min samples failing shortly after the onset of plastic deformation with less than 10% elongation at failure. The highest values of strain at failure were observed for the 20 min + fibrin (49.6% ± 2.2) and 20 min + fibrin at 37 °C (38.0% ± 4.5) samples, both within range of natural ACL tissue [29].

**Figure 4:**
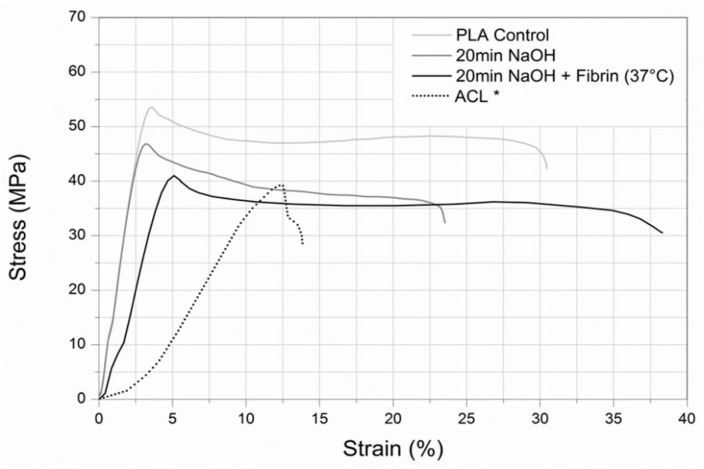
Overlay of the stress-strain profiles from a control, 20 min NaOH-treated sample, and 20 min NaOH-treated sample with fibrin at body temperature. Dotted lines indicate the stress-strain profiles of the ACL for comparison. Data adapted with permission from Butler, et al., J. Biomech. 1986, 19, 425–432. [29]. DOI: 10.1016/0021-9290(86)90019-9, Copyright Elsevier 2022.

The elastic modulus for the PLLA was consistent (2542 MPa ± 23) for all of the samples tested (*n* = 80) at room temperature. Samples were also tested at 29 °C and 37 °C to see the effects of temperature on the modulus, yielding values of 1884 MPa ± 39 and 1636 MPa ± 70, respectively. Figure 4 displays the percent strain at yield stress for the samples, with an average value of 3.1% ± 0.08 at room temperature and 5.1% ± 0.12 at 37 °C.

For the yield and maximum stress values displayed in Figure 3, it is theorized that the increase in surface roughness observed from past studies for the 5 and 10 min samples create friction between fibers during elastic deformation, leading to a subtle increase in strength when compared to the smooth surface morphology of the control and the (relatively) smooth surface of the 1 min samples [9]. The maximum stress values of the 1, 5, and 10 min samples are believed to be influenced by strain-induced crystallization, resulting in an increase in strength. This is thought to be caused by the hydrolytic degradation by NaOH and the subsequent lowering of the molecular weight on the PLLA fiber surface, leading to crystallization of the shorter chains primarily on the surface during strain. In terms of simple crystallization kinetics, a shorter polymer chain (lower molecular weight) can align into ordered lamellae more easily and rapidly than a longer chain. This is supported by a previous study, in which differential scanning calorimetry (DSC) data of electrospun PLLA fibers subjected to 20 min of hydrolysis exhibited a higher degree of crystallinity than an untreated sample [9].

There appears to be a combination of factors resulting in an ideal response or “sweet spot” in this study, where increased strength caused by decreased molecular weight (due to hydrolysis) is not affected by reduced strength caused by reduced fiber diameter (also due to hydrolysis). The 5 min and 20 min + fibrin samples both display these properties and are both within the range of the natural ACL and graft tissue for yield/maximum stress. The 5 min sample has higher stresses (yield and maximum) and greater percent elongation at failure than all of the other NaOH-treated samples. The 20 min + fibrin sample also displays a maximum stress higher than the yield stress, believed to be caused by a combination of strain-induced crystallization kinetics and the large extensibility of fibrin. However, the percent elongation at failure for the 20 min + fibrin samples is the highest out of all the samples tested, and it is within the range of natural ACL tissue. It is theorized that the addition of fibrin on the surface of the fibers fortifies the reduced diameter caused by the 20 min NaOH-treated PLLA, effectively negating the effects of lowered yield and maximum stresses and the strain at failure when compared to the 20 min sample without fibrin. 

The elastic modulus values of the PLLA are almost 4 times higher than the values of the patellar tendon (643 MPa ± 53) and between 7–40 times higher than the various ligament data (65–345 MPa), as seen in Supplementary Figure S1. The 37 °C sample is approximately 2.5 times higher than the patellar tendon; however, it indicates a 36% reduction in modulus compared to samples tested at room temperature. Supplementary Figures S2 and S3 of tested scaffolds display (the percent strain at yield stress and % failure strain at room temperature and at 37 °C) with values that are approximately 4 and 2.5 times lower than the yield strain values for the patellar tendon, respectively.

In the present case of using aligned PLA nanofibers to replicate the mechanical function of an ACL, the modulus and strain at yield stress are outside of the range of values found in the human ligament and ligament-replacement graft tissue. Altering the molecular weight of the polymer could further reduce the modulus and subsequently increase the strain at yield stress, although the effects of NaOH and fibrin treatments would have to be re-evaluated and optimized.

To visually understand the overall effects of the various surface treatments and temperature on the mechanical behavior of the PLLA fibers, Figure 4 displays the stress-strain profiles of a control, 20 min NaOH-treated sample, and 20 min NaOH-treated sample with fibrin at body temperature. A representative stress-strain profile of an ACL was included for comparison. The stepwise process of electrospinning a highly aligned PLLA nanofiber mat, followed by a 20 min NaOH hydrolysis treatment and the immobilization of fibrin on the hydrolyzed surface of the fibers results in a mechanical profile more similar to human ligament tissue than unmodified PLLA.

The high degree of fiber alignment achieved during the unidirectional electrospinning technique minimized the inter-fiber spacing considerably when compared to unaligned electrospun fibers; however, inter-fiber spacing still exists. The macroscopic (bulk) mechanical properties of the fibers tested in uniaxial tension may therefore vary from the microscopic mechanical properties of an individual electrospun fiber tested in uniaxial tension. Previous studies on aligned electrospun polymer fibers propose that the parallel stacking of the fibers causes behavior comparable to a thin film [3]. However, due to the resulting porosity, the samples will have a smaller cross-sectional area than the area obtained through measurement, yielding potentially different results. Another reason can be attributed to differences in the testing parameters of this study compared to studies of natural ligament tissue. The strain rate used by Noyes et al. [9] to study ACL behavior under uniaxial tension was 30 mm/s.

### 3.2. Fiber Alignment, Diameter, and Morphology

**Figure 5:**
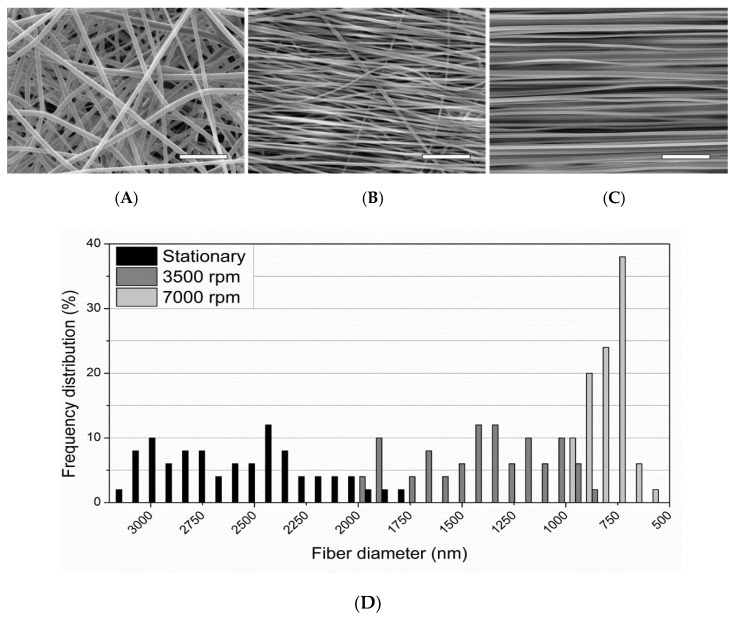
SEM images of electrospun PLLA fibers using the following uptake rates: (**A**) stationary, (**B**) 3500 rpm, (**C**) 7000 rpm. Magnification = 1000×, Scale bars = 25 µm. (**D**) Frequency distribution of electrospun PLLA fiber diameter at 0 rpm (stationary), 3500 rpm, and 7000 rpm uptake rates measured from SEM images.

Fibers were collected on three uptake conditions: stationary (0 rpm), 3500 rpm mandrel rate, and 7000 rpm mandrel rate. SEM images exhibit nanofibrous morphology for all conditions with varying degrees of inter-fiber spacing, diameter, and alignment (Figure 5). The stationary sample has relatively straight fibers that were randomly oriented with an average diameter of 2608 ± 353 nm (Figure 5A). The 3500 rpm sample shows a more ordered alignment and orientation perpendicular to the axis of mandrel rotation, reduced inter-fiber spacing compared to the stationary sample, and an average fiber diameter of 1396 ± 312 nm (Figure 5B). As the uptake rate increases to 7000 rpm, the fibers achieve a highly-aligned morphology perpendicular to the axis of mandrel rotation. The inter-fiber spacing is significantly reduced in comparison to the stationary and 3500 rpm samples. Similarly, the average fiber diameter is reduced to 760 ± 96 nm with greater regularity (Figure 5C).

These findings indicate that increased uptake rates for electrospun PLLA result in increased fiber order and alignment, and the rates are inversely proportional to fiber diameter and spacing. Thomas et al. [3] reported similar findings with electrospun PCL. They postulated that the fiber diameter decreases and alignment increases due to the fibers stretching to a greater degree at higher uptake rates.

As mentioned above, the average fiber diameters ranged from 760–2608 nm depending on the uptake condition. The range of fiber diameters within a specific uptake condition is also uptake-rate dependent. Figure 5 also shows the frequency distribution of fiber diameters for the electrospun PLLA at the different uptake conditions. Increased uptake rates result in decreasing distributions of the fiber diameters. This is shown with the stationary, 3500 rpm, and 7000 rpm rates and their standard error of mean (S.E.M.) (*n* = 50) distributions of ±50, ±44, and ±14, respectively.

In order to facilitate a fiber diameter mimetic of the 50–500 nm diameter collagen bundles, the PLLA electrospun at the 7000 rpm rate was subjected to 0.25 M NaOH hydrolysis treatments of 1, 5, 10, 20, and 40 min time intervals. Table 1 shows the average diameter and reduction in cross-sectional fiber area because of the NaOH treatments. The 1, 5 and 10 min treatments all had fiber diameters above the upper limit of the collagen bundles. The two treatments at 20 and 40 min produced fibers that fall within the upper range of the collagen bundles with average diameters of 457 ± 89 nm and 399 ± 92 nm, respectively. However, the 40 min fibers were found to be very brittle and would separate easily during handling, while the 20 min samples maintained their structural integrity more similarly to the untreated samples.

The final cross-sectional area of the fibers was shown to be dependent on NaOH treatment time, with the percent reduction in area following a logarithmic trend as treatment time was increased (Supplementary Figure S4).

**Figure 6:**
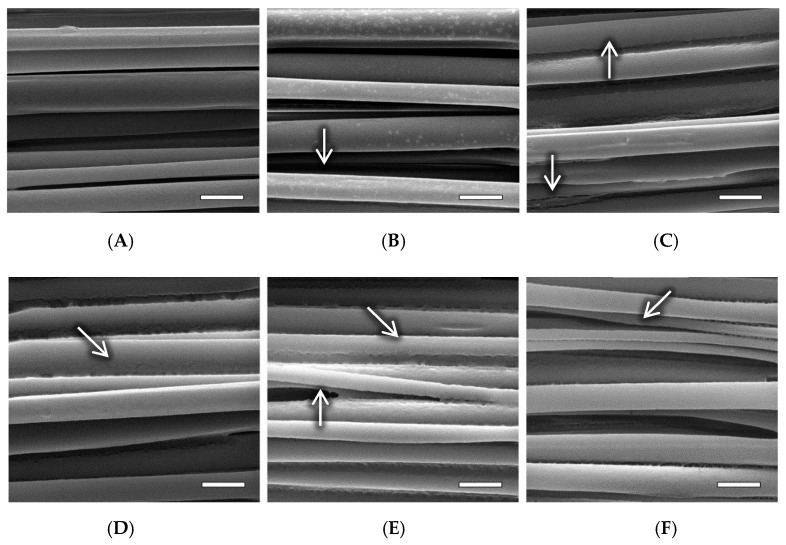
SEM images of electrospun PLA fibers subjected to the following NaOH treatment times: (**A**) 0 min (control); (**B**) 1 min, arrow indicates minor surface roughness; (**C**) 5 min, arrows indicate visible surface roughness and pitting at the fiber interface; (**D**) 10 min, arrow indicates interface pitting; (**E**) 20 min, arrows indicate significant surface roughness and pitting at the fiber interface; (**F**) 40 min, arrow indicates significant pitting and visibly decreased fiber diameter. Magnification = 50,000×. Scale bars = 1 µm.

The various NaOH treatments also had an effect on the surface morphology of the fibers, as observed in Figure 6. The control sample (Figure 6A) has a smooth and continuous surface morphology. There are some small cracks (<100 nm) present at certain points on the fibers that are barely visible, believed to be caused by expansion/contraction of the polymer and subsequent cracking of the gold-palladium coating during sample preparation, or by thermal damage from the electron beam during SEM imaging. The fibers display no pitting or altered shape/thickness along the length of the fibers. The 1 min sample displays relatively smooth fiber morphology with a very minor amount of pitting and surface roughness, indicated by the arrow in Figure 6B. There are lightly colored spots on the fibers that were found throughout all of the 1 min samples, believed to be points of increased surface roughness. When observed at the visible edge of a fiber, there appears to be a subtle dip in surface topography. Unfortunately, attempts at viewing the spots at a higher magnification were not possible due to thermal damage caused by the electron beam on the polymer when magnification was increased. The 5 and 10 min samples in Figure 6C,D, respectively, exhibit more pronounced degradation compared to the control and 1 min samples. A higher degree of surface roughness can be observed, and there is visible pitting at the interface between fibers, indicated by the arrows. The 20 and 40 min samples display a pronounced degree of surface roughness and interface pitting, as indicated by the arrows in Figure 6E,F, respectively. Additionally, a reduced overall fiber diameter is visibly apparent when compared to the control sample. These data presented indicate that obtaining PLA nanofibers via unidirectional electrospinning with a mandrel rotational velocity of 24.6 m/s, followed by a 20 min treatment in 0.25 M NaOH solution will result in a polymer matrix with fiber alignment and diameter mimetic of native collagen bundles found in human ligament tissue.

### 3.3. Analysis of Crystallinity

The results of the DSC analysis are displayed in Table 2. The various morphologies had little effect on the melting temperatures; however, the melting enthalpy was significantly reduced with the solution cast compared to the as-received sample. Melting enthalpy and subsequent crystallization increased when the PLLA solution was electrospun into randomly oriented fibers. Crystallinity was further increased when the fibers were collected on the 7000 rpm rotating mandrel. The reduced diameters of the aligned fibers indicate a significant degree of fiber stretching, and subsequently, strain-induced crystallization. The 20 min NaOH-treated aligned fibers displayed a slight increase in crystallinity compared to the untreated aligned fibers, presumably due to polymer chain scission caused by the NaOH hydrolysis and the subsequent reduction in molecular weight on the fiber surface.

### 3.4. Swelling Data

**Figure 7:**
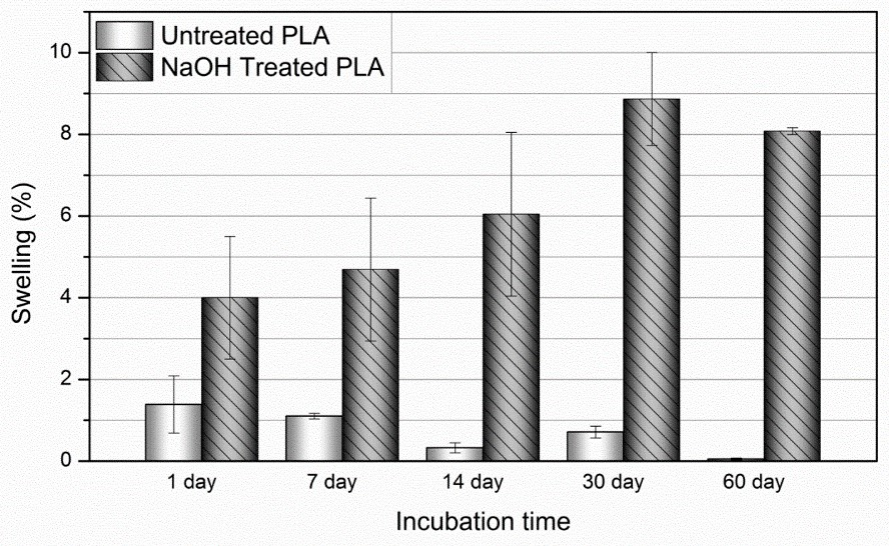
Average percent swelling of the PLLA fibers in the untreated and NaOH-treated conditions over 60 days. Values expressed as mean ± S.E.M.

Figure 7 displays the percent swelling of the PLA fibers in the untreated and NaOH-treated conditions over a 60-day period. Samples in the untreated condition displayed minimal swelling (0.07–1.1%), presumably due to the high hydrophobicity of PLA. Conversely, the NaOH-treated samples displayed modest swelling (4.04–9.2%) due to their increased surface roughness and surface carboxyl groups. It is presumed that this effectively reduces hydrophobicity and increases PBS uptake.

### 3.5. Immobilization of Fibrin

**Figure 8:**
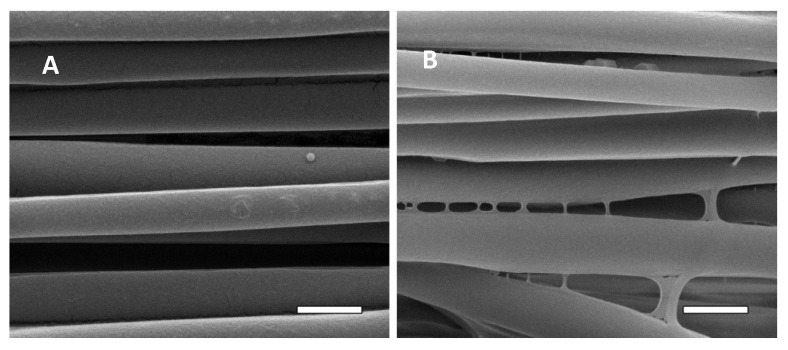
SEM images of electrospun PLLA fibers subjected to fibrin immobilization in the: (**A**) untreated condition, arrow indicates a particle of (presumably) fibrin found randomly throughout the sample; (**B**) 20 min NaOH treatment, arrows indicate connecting membranes of fibrin. Magnification = 50,000×. Scale bars = 1 µm.

Samples of the 7000 rpm PLA in the untreated and 20 min NaOH treatment conditions were subjected to surface immobilization of fibrin. As can be seen in Figure 8, there is a significant difference in the surface morphology of the untreated PLLA (Figure 8A) and the NaOH treated sample (Figure 8B). The surfaces of the untreated fibers are smooth, continuous, and closely resemble the control sample in Figure 6A, with the exception of particles randomly scattered along the surface, presumably comprised of fibrin (indicated by an arrow in Figure 8A). Conversely, the NaOH-treated sample displays a different morphology from the untreated sample with the added fibrin, as well as the sample with the same NaOH treatment in Figure 6E. The pitting and rough surface topography seen in the samples with the same 20 min NaOH treatment has been eliminated and is generally smooth with an apparently confluent superficial layer of fibrin. Another indicator of confluence is the presence of connecting membranes at the interface between fibers, indicated by the arrows in Figure 8B. It is presumed that the highly hydrophobic nature of the untreated PLLA may inhibit effective adhesion of fibrin, but when hydrolytically degraded by NaOH, the subsequent addition of the carboxyl groups and increase in surface roughness results in an increased binding affinity of fibrin. These findings correspond to the aforementioned studies indicating the binding affinity of fibrin to surface carboxyl groups via hydrolytic degradation Scheme 1, Supplementary Figure S5. There is a pronounced decrease in the intensity of the ester group peak due to NaOH treatment, and additional peaks for protein appeared due to amide carbonyl and amino groups in the fibrin-treated sample in the infrared spectra (Supplementary Figure S5).

### 3.6. In Vitro Human Mesenchymal Stem Cell (hMSCs) Studies

Alternative ACL replacement grafts using non-resorbable materials have been investigated over the last 40 years with limited success, due to in part to difficulties with implementing an effective and durable fixation point for integrating the synthetic graft to bone [31,32]. Because enthesis between graft and bone is essential for musculoskeletal motion, the fixation of any grafts is crucial in the repair of injuries to ligaments and tendons [33].

In order to determine the state of osteogenic differentiation of hMSCs, they must be assessed for changes in morphology, protein production, and proliferation over time. At the onset of differentiation, hMSCs slowly up-regulate genes indicative of an osteogenic phenotype. Furthermore, as hMSCs differentiate into osteoblasts, their rate of proliferation should slow [34,35]. ALP plays an important role in the mineralization of bone tissue. As differentiation proceeds, ALP activity rises and cleaves organic phosphates to produce free, inorganic phosphates [36,37]. ALP activity has become an important indication of osteogenic differentiation [34,38].

To address how the surface characteristics can affect the attachment and proliferation of hMSCs, a previous study involved fabricated titanium, aluminum, and HA ceramics with grain sizes of 1500, 200, and 50 nm [39]. hMSCs were seeded on these constructs and assessed for attachment at 1 and 4 h as well as proliferation at days 1, 3, and 7. Results indicated that attachment was greatest on the 1500 nm grain-size constructs and the lowest on the 50 nm grain-size constructs. However, cellular growth proceeded the quickest on the 200 nm constructs and the slowest on the 50 nm constructs. Taking these results into account, it was demonstrated that surface characteristics can affect the short-term behavior of hMSCs. Therefore, the surface characteristics of the PLLA mats must be considered when assessing cellular behaviors.

**Figure 9:**
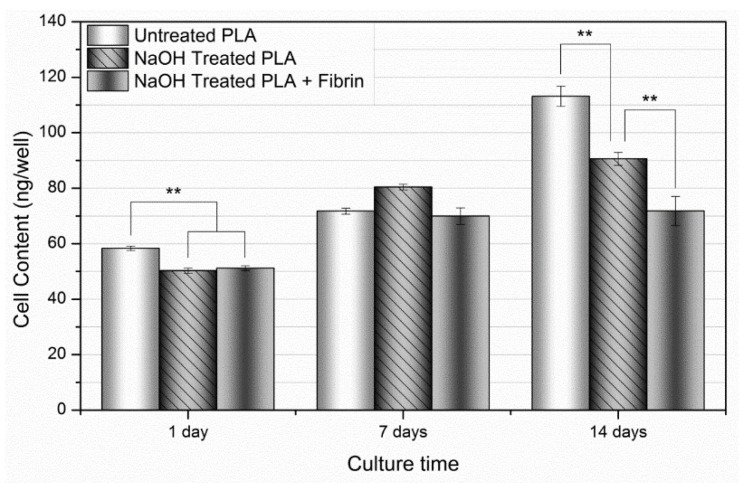
Cellular proliferation over 14 days. Over the 14-day time frame, there was an overall increase in proliferation at day 7, followed by a stepwise increase manifesting at day 14. Values expressed as mean ± S.E.M. (** *p* < 0.01).

Three conditions of the PLA fiber mats (untreated, 20 min NaOH, and 20 min NaOH + fibrin) were prepared for the evaluation of the hMSC response over 1, 7, and 14 day time intervals. As mentioned earlier, withdrawal from the cell growth cycle ifs an important indication of the onset of differentiation towards an osteogenic lineage from an undifferentiated state [35,40]. To determine their proliferative rate over time, hMSCs were cultured on three conditions of the PLLA fiber mats (untreated, 20 min NaOH, and 20 min NaOH + fibrin), and their long-term proliferation was assessed over 14 days (Figure 9). By day 14, cellular proliferation had progressed in a fashion highlighting a decrease in proliferative rate based on modifications to the PLLA fiber mats. Proliferation proceeded the quickest on the untreated PLLA fiber mats, approaching a cellular DNA content of 113.13 ± 3.66 ng/well, with cells cultured on 20 min NaOH-treated PLA mats displaying slightly less proliferation over time. The addition of fibrin onto the 20 min NaOH-treated PLLA mat further slowed proliferation, showing little increase in cellularity between days 1 and 14. Considering that proliferation is known to plateau at the onset of differentiation, it is possible that NaOH treatment modified the PLLA surface properties in a manner that facilitated osteogenic differentiation, and that the addition of fibrin does so further.

**Figure 10:**
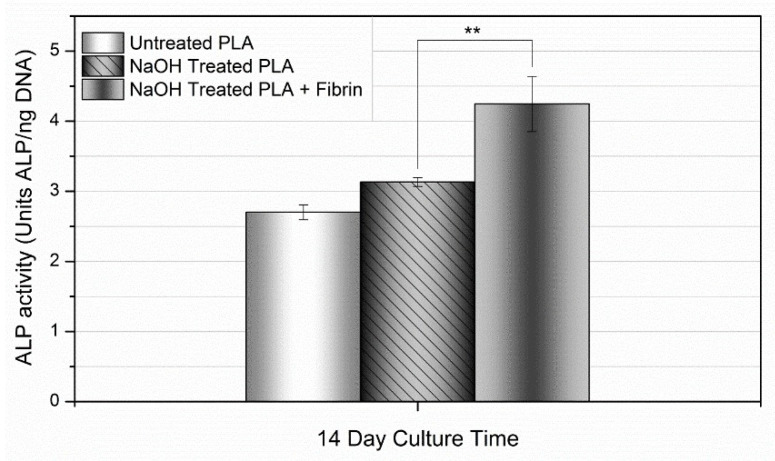
ALP activity over 14 days. At day 14, ALP activity was greatest in the NaOH-treated PLLA + fibrin samples. Values expressed as mean ± S.E.M. (** *p* < 0.01).

As mentioned earlier, ALP is an important protein responsible for cleaving organic phosphates to produce free, inorganic phosphates for the process of mineral deposition. In order to assess mineralization, hMSCs were cultured on the modified PLA fiber mats for up to 14 days, following which physical ALP activity was determined utilizing a quantitative biochemical assay (Figure 10). These values are normalized by DNA content as a means in order to assess the activity of each individual cell as opposed to total ALP activity. By day 14, the 20 min NaOH + fibrin condition exhibited statistically greater ALP activity relative to the other conditions.

**Figure 11:**
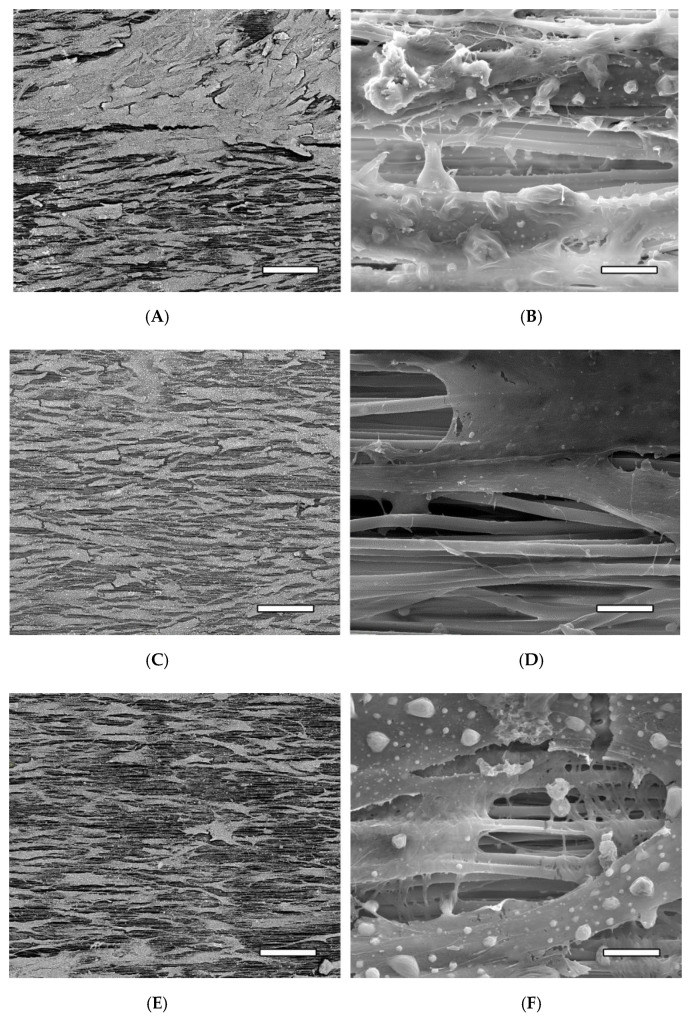
SEM images of the hMSCs on the PLLA samples at day 14 at 250× in BSE mode (left images) and 50,000× in SE mode (right images). (**A**,**B**) Untreated PLLA, (**C**,**D**) 20 min NaOH-treated PLLA, and (**E**,**F**) 20 min NaOH-treated PLLA + fibrin. Scale bar (left images) = 200 µm, (right images) = 5 µm.

SEM images of the hMSCs on the various PLLA samples at day 14 are displayed in Figure 11. Figure 11A displays the untreated PLLA sample at low magnification; a fairly confluent layer of cells can be seen on the sample with individual cells displaying an elongated and spindle-like morphology aligned in the general direction of the PLLA fibers. A higher magnification (50,000×) image of the same sample is displayed in Figure 11B. The PLLA fibers are visible, with the cells exhibiting finger-like projections extending towards the fibers. Figure 11C displays the 20 min NaOH-treated sample with a visible cell density slightly less than what can be seen on the untreated sample and with a higher degree of cell alignment but similar cell morphology. The higher magnification image corresponding to the 20 min NaOH sample is shown in Figure 11D. There is not a significant amount of discernible difference at high magnification between the untreated sample and the 20 min NaOH sample. Figure 11E shows the fibrin-coated NaOH treated sample. There is a clear reduction in cell density on the surface compared to the other samples at that magnification. The morphology of some of the cells appears to be slightly less elongated or spindle-like, adopting a more rectangular or cuboidal shape. The high-magnification image shows small interconnecting strings of fibrin between the PLLA fibers and more pronounced cellular processes integrating with the fibers and fibrin.

The various images in Figure 11 correlate to the quantitative data obtained from the analysis of proliferation and the ALP study: the untreated sample had a visibly higher quantity of cells on the surface with a diminishing amount for the NaOH treated and the NaOH + fibrin treated samples, respectively. It is also interesting to note that the cells spread and oriented themselves along the direction of the fibers. This is of importance in the present case of an engineered ligament due to the fact that an aligned orientation of cells and collagen fibers is found in native ligament tissue; subsequent differentiation and ECM production of the hMSCs on the scaffold could theoretically follow the same morphology due to the presence of the aligned PLLA fibers.

The addition of fibrin led to even further enhanced ALP activity. This is a possible result of the cell-ECM interactions taking place due to integrin-mediated binding mechanisms between the hMSCs and fibrin.

Cells interact with the ECM via various integrin and non-integrin binding mechanisms, in which different cell types will recognize and bind to cellular adhesive ligands transcribed into ECM proteins, such as collagen, fibronectin, vitronectin, and laminin. Cellular adhesive ligands are signaling mechanisms built into various ECM proteins and are designed to bind to transmembrane receptors (integrins) to induce conformational changes in the receptor, leading to various cellular responses. Depending on the type of integrin signaling molecules present within the ECM, cellular behaviors such as proliferation, growth, migration, and differentiation will be modified. In this regard, previous studies have demonstrated osteogenic differentiation driven by integrin-mediated binding mechanisms both with and without the presence of osteogenic supplement media [41,42].

## 4. Conclusions

Mechanical and morphological studies of unidirectionally electrospun and fibrin-immobilized PLLA fiber mats with various stepwise surface treatments were reported. Induced hydrolytic degradation of the fibers in 0.25 M NaOH solution followed by immobilization of fibrin on the hydrolyzed fiber surfaces was shown to significantly affect the mechanical properties of hybrid scaffolds. NaOH hydrolysis treatment displayed significant surface roughness with pitting at the interface between fibers. DSC data indicated the degree of crystallinity of the PLLA followed the following trend: solution cast thin film < unaligned electrospun fibers < aligned electrospun fibers < aligned electrospun fibers treated by NaOH hydrolysis < as-received PLLA pellets. Fibrin was immobilized on the surface of the electrospun PLLA; SEM images show a confluent layer of fibrin on the hydrolyzed samples compared to random fibrin particles randomly found on the surface of the untreated PLLA fibers.

The preliminary proliferation and differentiation response of the hMSCs in vitro was quantitatively assessed using Picogreen dsDNA and ALP assays and qualitatively assessed using SEM. It was found that treatment with NaOH and fibrin immobilization enhanced the osteogenic differentiation of hMSCs. Additionally, SEM images indicated an aligned orientation of cells along the direction of the fibers in all samples. Overall, the present study indicated that unidirectional electrospinning of PLLA at a rotational velocity of 26.4 m/s produced highly aligned nanofibers that, when subjected to a 20 min hydrolysis treatment in 0.25 M NaOH solution and subsequent immobilization of fibrin on the fiber surface, produced a scaffold with increased in vitro bioactivity and which was mimetic in size and morphology of collagen bundles found in ligament tissue.

## Figures and Tables

**Table 1:** NaOH treatment effects on 7000 rpm PLA nanofibers.

NaOH Treatment Time (min)	Average Diameter (nm) ± S.E.M *	Reduction in Fiber Area (%) ± S.E.M *
0 (control)	760 ± 13.6	-
1	700 ± 13.6	13.6 ± 3.4
5	601 ± 11.1	36.4 ± 2.3
10	564 ± 12.3	43.5 ± 2.3
20	457 ± 12.5	62.5 ± 2.1
40	399 ± 13.1	70.9 ± 2.0

* *n* = 50.

**Table 2:** Differential scanning calorimetry (DSC) data of various PLA morphologies.

Sample	As Received (Pellet)	Solution Cast Thin Film	Espun Fibers (Random)	Espun Fibers (Aligned)	Espun Fibers (Aligned, 20 min NaOH Treated)
Melting Enthalpy (J/g)	60.58	34.06	41.54	54.14	54.62
Melting Temperature (°C)	178.72	174.93	174.74	174.17	174.55
Crystallization (%)	80.16	45.07	54.97	71.64	72.28

**Figure A1:**
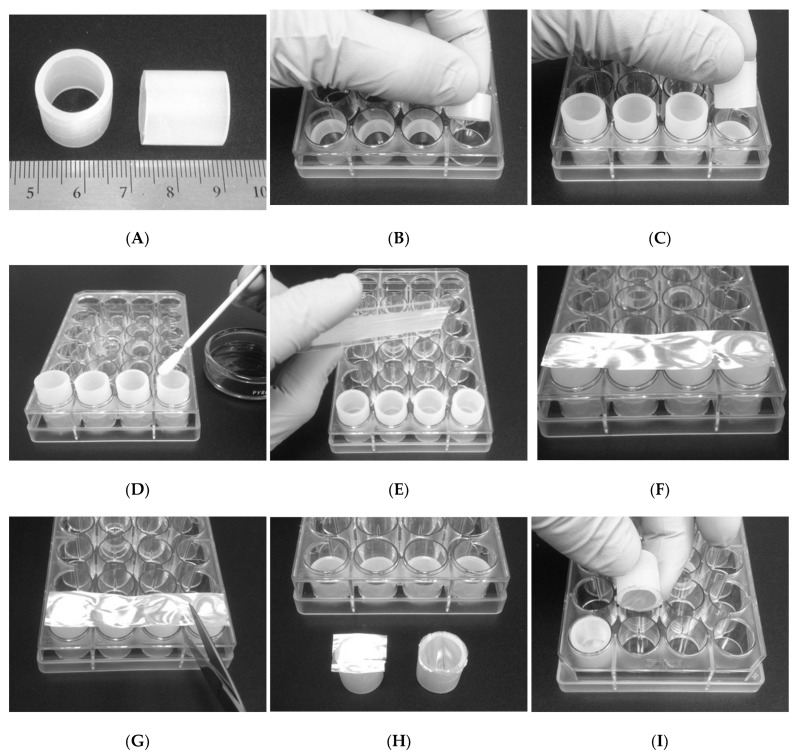
(**A**) Modification of 24-well tissue culture plates for sample fixation. (**B**) Sections of PE tubing Figure 10. mm risers at the bottom of the well plate used as a frame. (**C**) Placement of the PE tubes from “A” into the frame. (**D**) Application of a biocompatible adhesive to the flat, polished end of the PE tubes. (**E**) Placement of a glass slide with an electrospun PLA sample on four pre-glued PE tubes. (**F**) Transfer of electrospun PLA sample to the glued ends of the four PE tubes. (**G**) Separation of the PLA-capped tubes. (**H**) PLA-capped tube before (left) and after (right) trimming of excess PLA. (**I**) Placement of the PLA-capped tube face-down into a new, sterile 24-well culture plate.

## Data Availability

The data presented in this study are available upon request from the corresponding author.

## Supplementary Materials

The following supporting information can be downloaded at: https://www.mdpi.com/article/10.3390/pharmaceutics14020277/s1, Figure S1: Elastic modulus of the aligned PLA fibers at various temperatures, compared to natural ligament/graft tissue. Values expressed as Mean ± S.E.M. (PT = patellar tendon, “Ligament” includes ACL, PCL, and LCL). Data adapted with permission from “Comparison of material properties in fascicle-bone units from human patellar tendon and knee ligaments”. by D. L. Butler, M. D. Kay, and D. C. Stouffer, J Biomech, vol. 19, pp. 425-32, 1986. DOI: 10.1016/0021-9290(86)90019-9 Copyright Elsevier 2022, Figure S2: Percent strain at yield stress of the aligned PLA fibers at various temperatures, compared to natural ligament/graft tissue. Values expressed as Mean ± S.E.M. (PT = patellar tendon, “Ligament” includes ACL, PCL, and LCL). Data adapted with permission from “Comparison of material properties in fascicle-bone units from human patellar tendon and knee ligaments”. by D. L. Butler, M. D. Kay, and D. C. Stouffer, J Biomech, vol. 19, pp. 425-32, 1986. DOI: 10.1016/0021-9290(86)90019-9 Copyright Elsevier 2022, Figure S3: Percent strain at failure of the aligned PLA fibers with various surface treatments, compared to natural ligament/graft tissue. Values expressed as Mean ± S.E.M, Figure S4: Percent reduction in area of 7000 rpm PLA nanofibers from various NaOH treatment times. Values expressed as Mean ± S.E.M. Percent reduction in area is dependent on NaOH treatment time and displays a logarithmic trend, r2 = 0.9816, Figure S5: Chemical surface variation of fibers treated with protein (C) and NaOH (B) and untreated (A).

## Appendix A. Sample Fixation to the Tissue Culture Plates

Polyethylene (PE) tubing (LLDPE Value-Tube PE58ANA, Advanced Technology Products, Milford Center, OH, USA) with 15.875 mm O.D and 12.7 mm I.D. was cut into 18 mm sections (Figure A1A). The dimensions selected resulted in the PE sections fitting snugly in the wells of a 24-well cell culture plate (M-353047, Beckton Dickinson and Co, Franklin Lakes, NJ, USA). One end of the tubes was ground flat and smooth using progressively finer grit sandpaper (240, 320, 400, and 600, respectively). The sections were washed with water, sonicated in a bath of acetone to remove any surface oils and ink product identification markings, followed by a 1 h soak in ethanol for sterilization. To create a frame to hold the samples during preparation, a 24-well plate was modified by inserting 10 mm sections of the PE tubing at the base of four of the wells on one side of the plate to act as risers (Figure A1B). Four PE sections were inserted into the modified 24-well plate at a time (polished side up) and a biocompatible adhesive (Mastisol, Ferndale Laboratories, Inc., Ferndale, MI, USA) was brushed onto the polished surface (Figure A1C,D). Sections (15 mm × 75 mm of the various PLA treatments were placed on a 25 mm × 75 mm glass microscope slide (held by static charge), aligned with the four PE tubes, and pressed firmly and evenly on the tubes. This resulted in the effective transfer of the PLA section to the row of PE tubes (Figure A1E,F). The PLA was then cut with scissors between the PE tubes (Figure A1G), and the separated tubes were removed from the well plate frame, individually “stamped” against a rubber surface to ensure complete adhesion of the PLA to the PE tube, and placed in a vacuum/desiccator to allow the adhesive to fully dry overnight. After the adhesive was dry, the PLA was trimmed around the PE tubes, then the tubes were placed in sterile 24-well culture plates (Figure A1H,I).
