# Supplementary material for: Uni-Directionally Oriented Fibro-Porous PLLA/Fibrin Bio-Hybrid Scaffold: Mechano-Morphological and Cell Studies
Source: Pharmaceutics. 2022 Jan 25;14(2):277. doi: 10.3390/pharmaceutics14020277 (PMC8879164; doi:10.3390/pharmaceutics14020277)
Supplement: Supplementary file 1 [file pharmaceutics-14-00277-s001.zip › pharmaceutics-1550282-supplementary.pdf]

pharmaceutics

Supplementary MATERIAL: Uni-Directionally Oriented Fibro-Porous PLLA/Fibrin Bio-Hybrid Scaffold: Mechano-Morphological and Cell Studies

Andrew F. Uehlin, Jeremy B. Vines, Dale S. Feldman, Elijah Nyairo, Derrick R. Dean and Vinoy Thomas

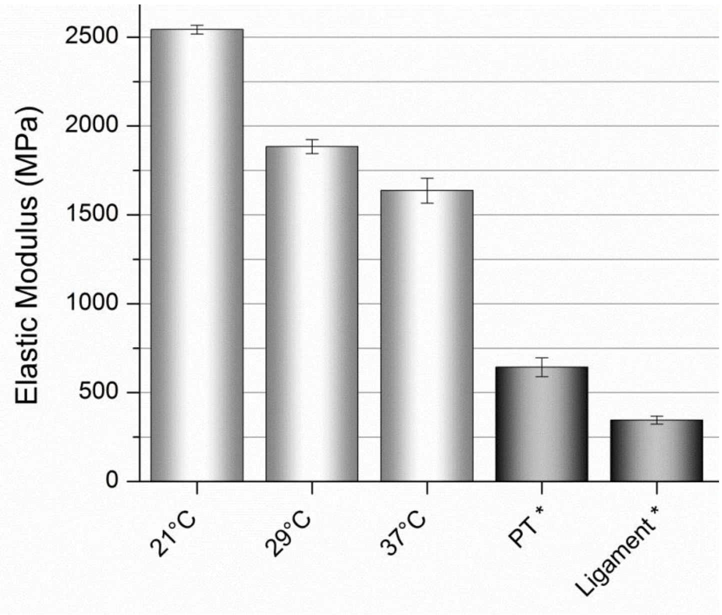

Figure S1. Elastic modulus of the aligned PLA fibers at various temperatures, compared to natural ligament/graft tissue. Values expressed as Mean ± S.E.M. (PT = patellar tendon, "Ligament" includes ACL, PCL, and LCL). Data adapted with permission from "Comparison of material properties in fascicle-bone units from human patellar tendon and knee ligaments." by D. L. Butler, M. D. Kay, and D. C. Stouffer, J Biomech, vol. 19, pp. 425–432, 1986. DOI: 10.1016/0021-9290(86)90019-9 Copyright Elsevier 2022.

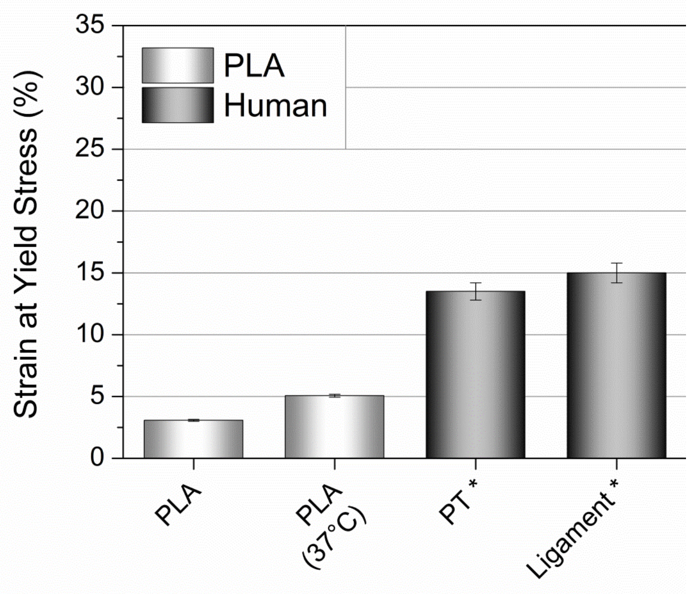

Figure S2. Percent strain at yield stress of the aligned PLA fibers at various temperatures, compared to natural ligament/graft tissue. Values expressed as Mean ± S.E.M. (PT = patellar tendon, "Ligament" includes ACL, PCL, and LCL). Data adapted with permission from "Comparison of material properties in fascicle-bone units from human patellar tendon and knee ligaments." by D. L. Butler, M. D. Kay, and D. C. Stouffer, J Biomech, vol. 19, pp. 425–432, 1986. DOI: 10.1016/0021-9290(86)90019-9 Copyright Elsevier 2022.

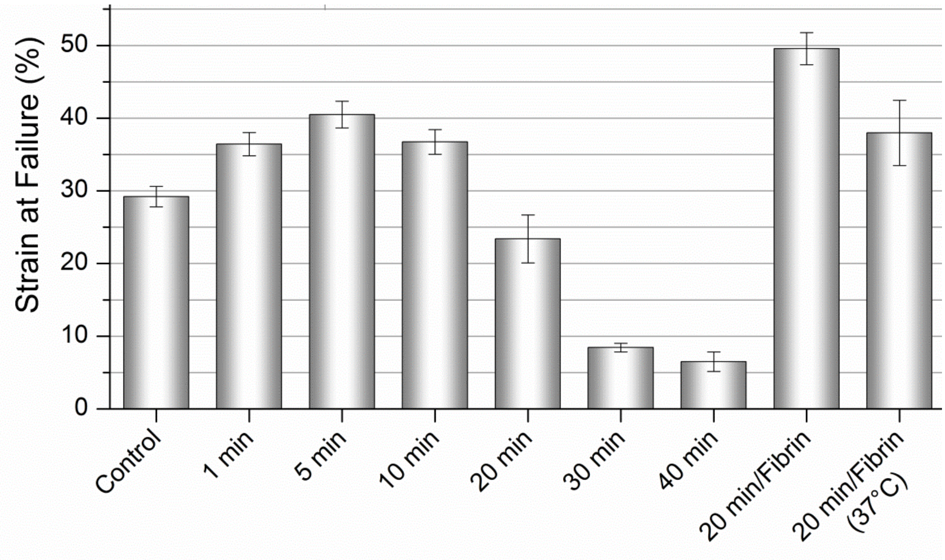

Figure S3. Percent strain at failure of the aligned PLA fibers with various surface treatments, compared to natural ligament/graft tissue. Values expressed as Mean ± S.E.M.

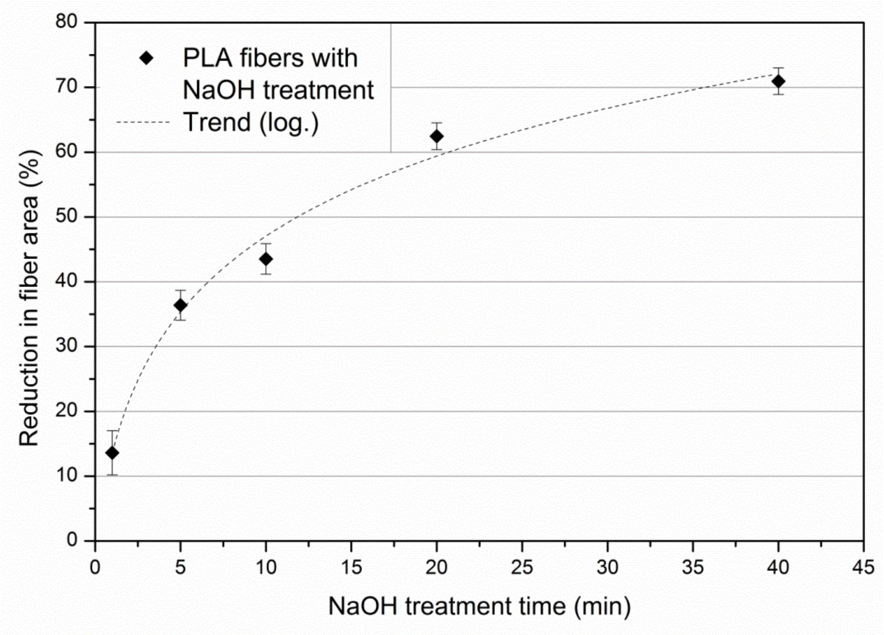

Figure S4. Percent reduction in area of 7000 rpm PLA nanofibers from various NaOH treatment times. Values expressed as Mean ± S.E.M. Percent reduction in area is dependent on NaOH treatment time and displays a logarithmic trend, r2 = 0.9816.

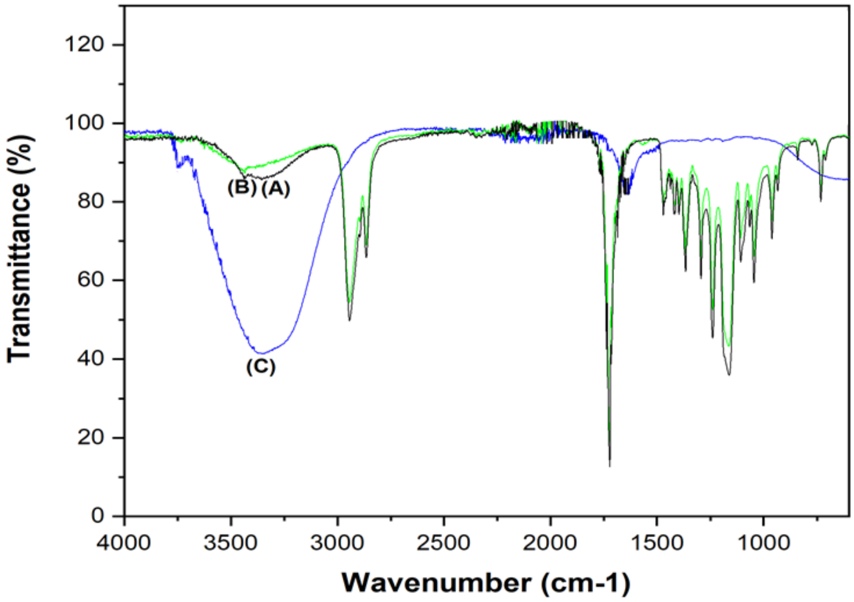

Figure S5. Chemical surface variation of fibers treated with protein (**C**) and NaOH (**B**) and untreated (**A**).
